# Transcriptome Dynamics of Human Neuronal Differentiation From iPSC

**DOI:** 10.3389/fcell.2021.727747

**Published:** 2021-12-14

**Authors:** Meltem Kuruş, Soheil Akbari, Doğa Eskier, Ahmet Bursalı, Kemal Ergin, Esra Erdal, Gökhan Karakülah

**Affiliations:** ^1^ Department of Histology and Embryology, Faculty of Medicine, Izmir Katip Çelebi University, Izmir, Turkey; ^2^ İzmir Biomedicine and Genome Center, İzmir, Turkey; ^3^ İzmir International Biomedicine and Genome Institute, Dokuz Eylül University, İzmir, Turkey; ^4^ Department of Histology and Embryology, Faculty of Medicine, Adnan Menderes University, Aydın, Turkey; ^5^ Department of Medical Biology and Genetics, Faculty of Medicine, Dokuz Eylül University, İzmir, Turkey

**Keywords:** iPSC-derived neuronal differentiation, transcriptome profiling, lncRNAs, coexpression, WGCNA

## Abstract

The generation and use of induced pluripotent stem cells (iPSCs) in order to obtain all differentiated adult cell morphologies without requiring embryonic stem cells is one of the most important discoveries in molecular biology. Among the uses of iPSCs is the generation of neuron cells and organoids to study the biological cues underlying neuronal and brain development, in addition to neurological diseases. These iPSC-derived neuronal differentiation models allow us to examine the gene regulatory factors involved in such processes. Among these regulatory factors are long non-coding RNAs (lncRNAs), genes that are transcribed from the genome and have key biological functions in establishing phenotypes, but are frequently not included in studies focusing on protein coding genes. Here, we provide a comprehensive analysis and overview of the coding and non-coding transcriptome during multiple stages of the iPSC-derived neuronal differentiation process using RNA-seq. We identify previously unannotated lncRNAs *via* genome-guided *de novo* transcriptome assembly, and the distinct characteristics of the transcriptome during each stage, including differentially expressed and stage specific genes. We further identify key genes of the human neuronal differentiation network, representing novel candidates likely to have critical roles in neurogenesis using coexpression network analysis. Our findings provide a valuable resource for future studies on neuronal differentiation.

## Highlights

• We provide an overview of the past and current advancements in iPSC-derived cell differentiation.
• We summarize the transcriptome during critical stages of iPSC-derived neuron differentiation.
• We identify the distinct characteristics of each stage, including coding and lncRNA genes.

## 1 Introduction

Increasing number of studies have highlighted that pluripotent stem cell (ESC/iPSC) technologies provide a notable platform to generate specific types of neuron from healthy and patient-derived iPSCs, *in vitro* models to elucidate the biological cues of neuronal development and the cellular/molecular basis of neurological disease (Chambers et al., 2009; Karumbayaram et al., 2009; Nizzardo et al., 2010; Lancaster et al., 2013; Chanda et al., 2014; Bardy et al., 2015; Akbari et al., 2019a). To generate neurons from iPSCs, it is crucial to utilize stepwise protocols that mimic the signaling and molecular events which occur throughout brain development *in vivo*. First attempts developed neuronal lineage with differentiation steps upon embryoid bodies formation (Gaspard et al., 2008). A few of the major barriers in this field are the purity, viability, maturity and functionality of iPSC-derived cells. Chambers and others showed that treatment by SMAD inhibitor during differentiation increases the efficiency of neuronal lineage generation in adherent culture conditions (Chambers et al., 2009). Other groups modified this protocol afterwards to further maturation and long-term culture. Following SMAD inhibition, neural precursor cells (NPC) were being enriched and expanded during neurogenesis (Shi et al., 2012). Moreover, increase in our knowledge about the coordination of brain development has permitted to develop specific regions of the brain *in vitro* (Muratore et al., 2014; Tao and Zhang, 2016; Kikuchi et al., 2017). More recently, the utilization of iPSCs and 3D cell culture systems added another dimension to the generation of organ-like structures, termed organoids, to dissect the molecular events during brain development (Lancaster et al., 2013; Paşca et al., 2015; Birey et al., 2017). Therefore, an integrative approach combining molecular biology and bioengineering approaches with computational biology methods has been implemented to overcome these limitations and generate reliable, functional *in vitro* models. These *in vitro* models allow to investigate the transcriptome dynamics and characteristic parameters of generated cells during neuronal specification.

In the last decades, genome-wide studies have revealed that mammalian tissue specific coding and non-coding RNAs (ncRNAs) play critical roles in the regulation of biological/developmental processes, such as lineage commitment, cell fate decision and organogenesis (Cabili et al., 2011; Hu and Shan, 2016; Perry and Ulitsky, 2016; Rosa and Ballarino, 2016; Pal and Rao, 2017). Transcriptome profile of pluripotent stem cell-derived neurons was obtained using RNA-seq data and utilized to improve differentiation of neurons (Wu et al., 2010; Lin et al., 2011; Hjelm et al., 2013; Stein et al., 2014). Analysis of gene expression dynamics in human iPSC-derived neurons provide a solid framework to study early neural developmental process, progenitor differentiation, distinct axonal development (Compagnucci et al., 2015; Grassi et al., 2020; Lindhout et al., 2020). Large-scale transcriptomics studies in bulk or single cell level tried to dissect quantitative changes in neurons gene expression and map the neurons to the temporal and spatial brain development based on transcriptome similarity (van de Leemput et al., 2014; Close et al., 2017; Tanaka et al., 2020). Long non-coding RNAs (lncRNAs) are a sub-type of ncRNAs with a length of more than 200 nucleotides that originate from coding and non-coding locations of the genome (Lee, 2012; Ulitsky and Bartel, 2013). In particular, lncRNAs participate in the patterning of cellular reprograming, maintenance of pluripotency, and specification of stem cells. In this regard, lncRNAs such as rhabdomyosarcoma 2-associated transcript (RMST), in interaction with other genes such as SOX2 (Ng et al., 2012; Ng et al., 2013), mediate neurogenesis, Pax6 upstream antisense RNA (PAUPAR) lncRNA and Pax6 co-regulate gene sets and recruit transcription coactivators that affect the growth of neural progenitor cells (Vance et al., 2014), the PNKY lncRNA maintains the neural stem cell pool (Ramos et al., 2015), and the lncRNA DNMT1-Associated Long Intergenic Non-Coding RNA (DALI) is expressed in the embryonic brain, where it governs the proper differentiation and specification of neurons and maturation of neuroblastoma cells (Chalei et al., 2014).

Previous studies showed a repertoire of 4,000–20,000 lncRNA genes are differentially expressed in different cell types of the human brain (Vance et al., 2014; Ramos et al., 2015). However, the relationship between lncRNAs and neural lineage commitment is yet not described in depth. Therefore, we investigated the transcriptome dynamics of lncRNAs along with the protein coding genes to address the challenge of elucidating the characteristic features of cells during different stages of neural differentiation from iPSCs. We further performed genome-guided *de novo* transcriptome assembly to predict high confidence lncRNA genes not found in previous annotations. Our main goal was to investigate the stage-specific expression and possible function of protein coding genes and lncRNAs over the course of iPSCs-derived neural differentiation, as well as to identify previously unannotated lncRNAs with potentially key roles in the process. Our study proposes potential functions of annotated and novel lncRNAs based on coexpression network and hub gene analysis, and provides a useful resource for further studies that examine the roles of lncRNAs in biological processes, such as mammalian development and neurogenesis.

## 2 Results and Discussion

### 2.1 *In Vitro* Differentiation of iPSCs Into Neurons Using Monolayer Culture Conditions

**FIGURE 1:**
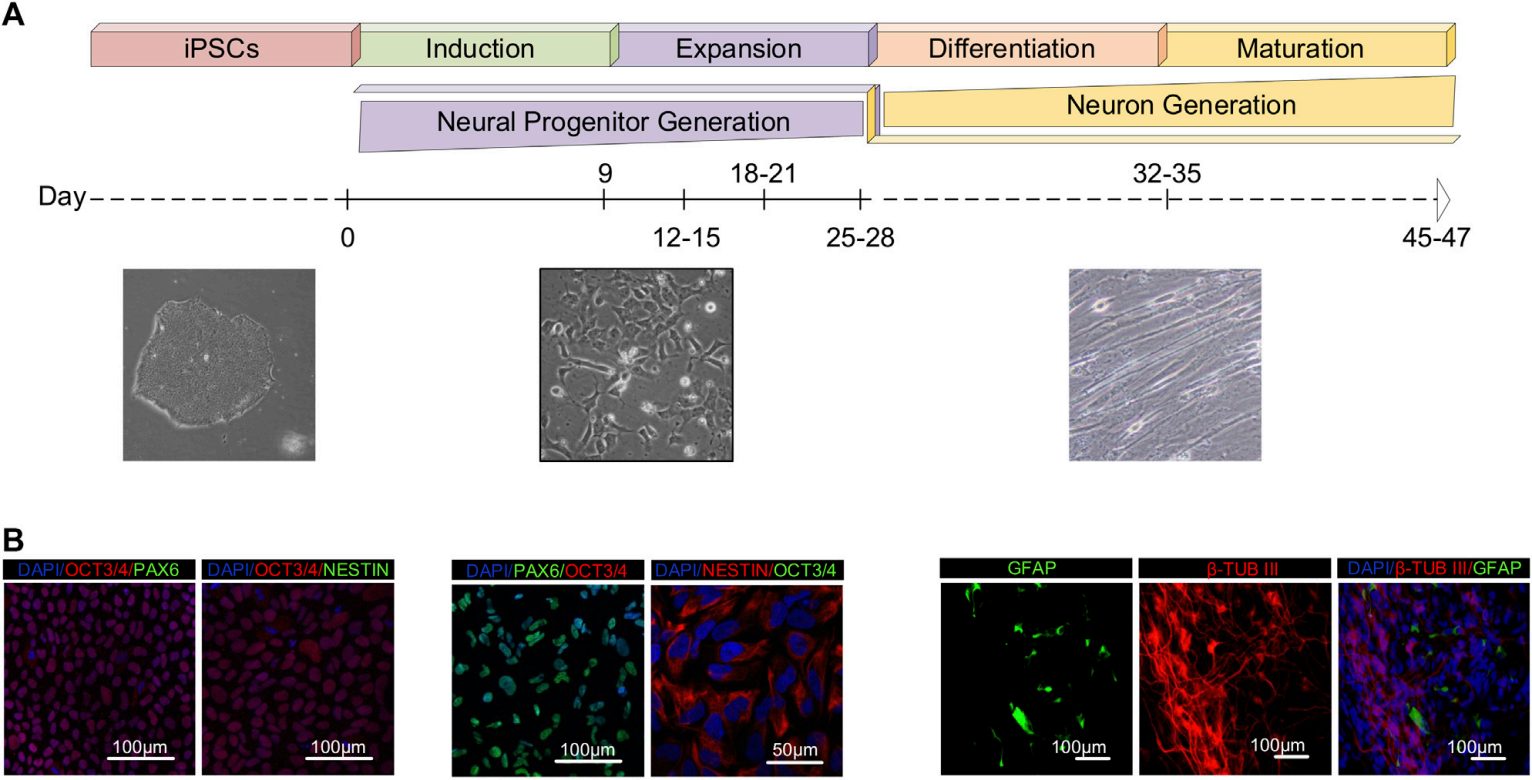
Generation and characterization of human iPSCs-derived neurons. **(A)** A schematic representation of the *in vitro* culture system used for stepwise differentiation of human iPSCs into neurons. Timeline and representative bright-field images of cell morphology during stages of differentiation from day 0 to day 45. **(B)** The samples for RNA-seq analysis were collected on day 0 (pluripotent), days 9–12 (induced progenitor), days 25–28 (expanded progenitor), days 32–35 (differentiated/neuronal precursor) and days 45–47 (mature neuron). Confocal images of the cells showing expression of the pluripotency marker (OCT3/4), neural progenitor markers (PAX6, NESTIN) and neural markers (GFAP, β-TUBIII). Nuclei were visualized with DAPI.

Human iPSCs cultured in feeder-free monolayer conditions were exposed to the neural induction medium, and subsequently re-plated in neuronal progenitor medium (NPM). Cells no longer exhibited pluripotent stem cell morphology during neural induction and progenitor expansion, and adopted an extended progenitor morphology instead (Figure 1A). Immunostaining analysis on day zero revealed that a majority of the cells were positive for the pluripotency marker OCT3/4, but not the neuronal progenitor markers PAX6 and NESTIN. Afterwards, we passaged and differentiated the iPSCs to generate neuronal progenitor cells. To assess neural progenitor (NP) generation, we first stained the cells with NP markers following differentiation. Between days 25–28 of neural progenitor generation, almost all cells were positive for the NP markers PAX6 and NESTIN. In addition, the OCT3/4 gene expression started to decrease, and a majority of the cells were negative for the pluripotency marker. Second, we passaged and cultured the NPCs as single cells for 21 additional days to further differentiate them, and analyzed the expression of markers indicating mature neuron cells on days 45–47. The maturation step resulted in the generation of class III β-TUBULIN-positive neurons with a very low proportion of GFAP-positive cells (Figure 1B). Taken together, this data indicates the iPSCs efficiently differentiated into neural cells.

### 2.2 Genome Guided *De Novo* Transcriptome Assembly and Transcriptome Profiling of iPSC Derived Neuronal Like Cells

**FIGURE 2:**
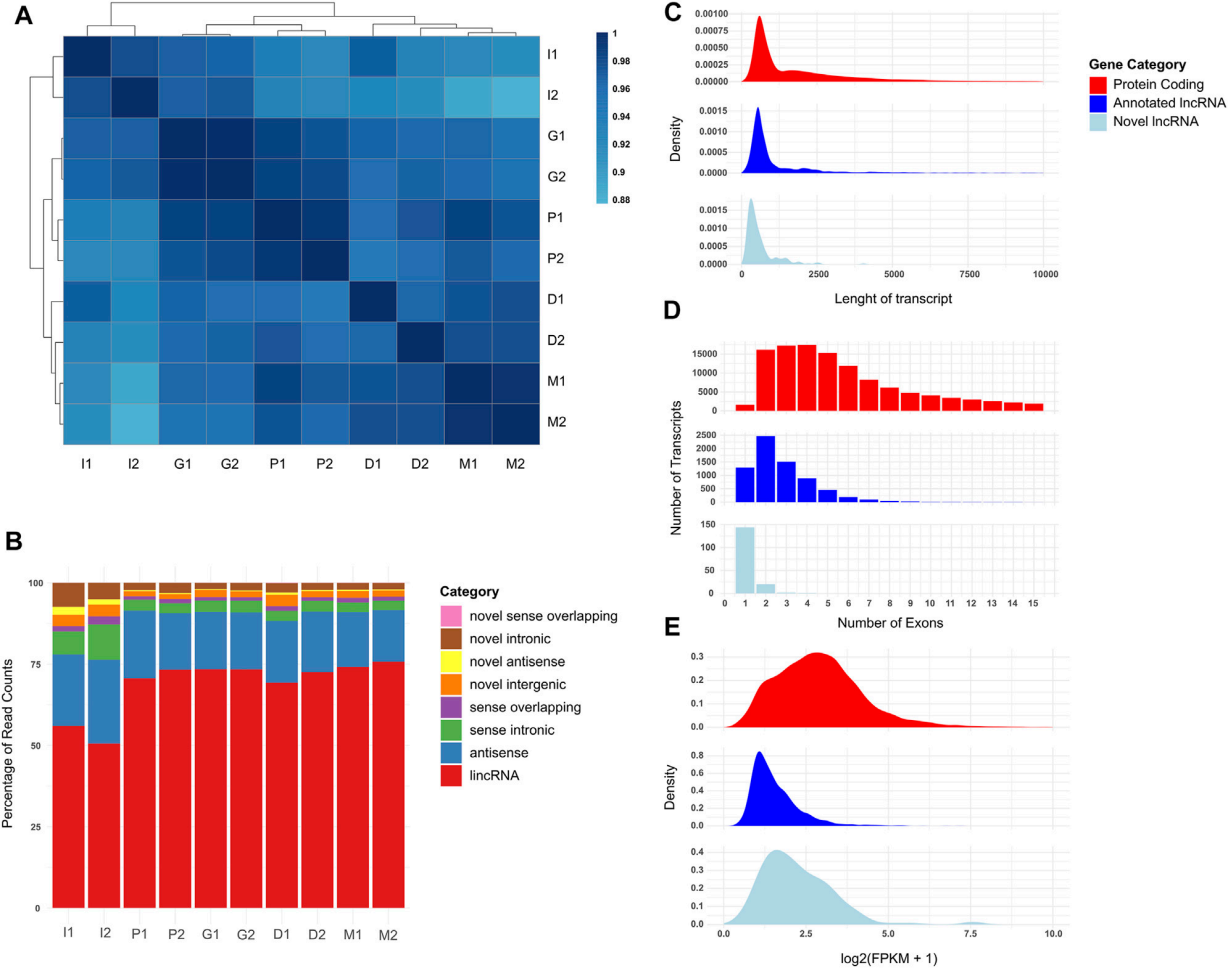
Comparison of the coding and non-coding transcriptome profiles of the cells during the neural differentiation process. I1 and I2 are iPSC samples, P1 and P2 are cells during neural induction, G1 and G2 are neural progenitor cells, D1 and D2 are cells undergoing neural differentiation, and M1 and M2 are mature neural cells. All transcriptome profiles are filtered for genes which display an FPKM value of at least 1.0 in both replicates of at least one biological condition. **(A)** Pearson correlation heatmap of samples clustered using hierarchical clustering. Cell colors indicate Pearson correlation values of the samples indicated in the row and column. Darker cells indicate higher correlation. **(B)** Stacked bar graph of long noncoding transcriptome profiles of the samples divided by percentage of RNA-seq counts sequenced per type. **(C)** Violin plot of protein coding and lncRNA expression values of the samples. **(D)** Density graph of genes by transcript length per gene type. **(E)** Histogram of genes by exon count in canonical transcript per gene type.

Following the characterization of the cells, we sought to understand the transcriptome profile of the cells using the RNA-seq technique. In addition to quantification of genes described in our annotation file (GENCODE GRCh38 human reference genome, Release 34), we used a robust novel lncRNA identification pipeline to identify whether any counts not aligned against annotated genes could have originated from previously unannotated transcripts (see Section 4). We have identified 354 high-confidence previously unannotated lncRNA candidates (herafter referred to as novel lncRNAs) (Supplementary File S1). After filtering lowly expressed genes from expression data, we used unsupervised hierarchical clustering of Pearson correlation values of the samples to generate a correlation heatmap (Figure 2A). The undifferentiated iPSCs (I1/I2) clustered together, while the neuron progenitor generation stages (P1/P2 and G1/G2) formed a separate cluster from the neuron generation stages (D1/D2 and M1/M2), as we expected during the neural differentiation process. Looking at the distribution of RNA-seq read counts across protein-coding, long non-coding, and other non-coding transcripts, we observed that long non-coding transcripts formed a very small fraction of total read counts in all samples (Supplementary Figure S1). We then looked at the distribution of reads across lncRNA transcript classes in more detail, divided into intergenic, antisense, sense overlapping, and sense intronic lncRNAs, further categorized as either annotated or novel (Figure 2B). Samples in different stages had varying read count distributions, suggesting a dynamic transcriptome profile, with annotated long intergenic non-coding RNAs (lincRNAs) comprising over 50% of the transcriptome in each stage, and a low percentage (<25%) of reads aligning to novel lncRNAs. We further characterized the distributions of the expressions of protein coding, annotated long non-coding, and novel non-coding genes in the samples (Supplementary Figure S2). We observed that lncRNA expressions are lower compared to protein coding gene expressions in all samples, with a few outlier lncRNA expressions being higher than protein coding genes in the same sample. Finally, we analyzed the length, exon distribution, and expression characteristics of protein coding, annotated lncRNA, and novel lncRNA genes expressed in our samples (Figures 2C–E), in order to determine whether the novel lncRNA characterization has been compatible with previously annotated lncRNAs. The analysis revealed protein coding genes as longer and with a higher exon count than both lncRNA categories (Figures 2C,D), as well as having higher expression (Figure 2E). Our results showed that our transcriptome sequencing and analysis have been consistent with expected findings.

We also inspected independent iPSC-derived neuronal differentiation datasets to observe whether reads in the datasets aligned to the same transcripts. To do so, we utilized two studies on iPSC-derived neuron transcriptomes that used a comparable sequencing depth and sequencing platforms, as well as ribosomal RNA depleted sequencing libraries (Burke et al., 2020; Solomon et al., 2021). We found that out of the 354 identified lncRNA candidates, 350 showed expression in at least one sample of the independent datasets, and 296 showed expression in one sample in both datasets (Supplementary Files S2, S3). When filtered for consistent expression across biological conditions, 89 of the lncRNAs showed an expression of 1 FPKM or higher in over 50% of the samples from a single biological condition in one dataset (Supplementary Figure S3), and 88 of them showed consistent expression in both datasets (Supplementary Figure S4). Our investigations into the independent datasets revealed that the majority of the novel lncRNA candidates were transcribed in other biological samples, and a number of the lncRNAs showed consistent transcription in similar biological processes across datasets.

### 2.3 Differential Expression of Protein Coding and lncRNA Genes Across Differentiation Steps

**FIGURE 3:**
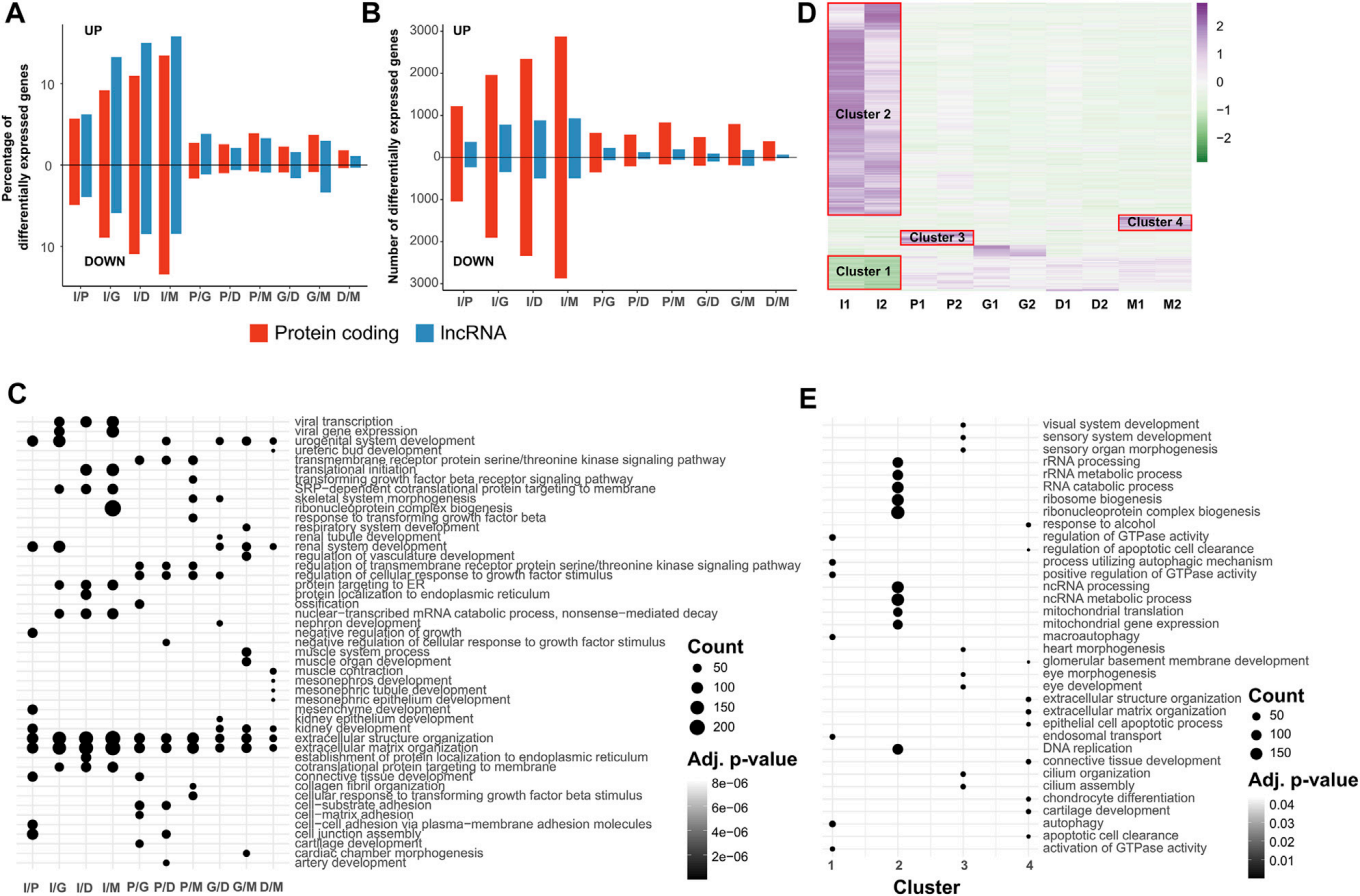
Genes showing differential or stage-specific expression during neural differentiation from iPSC cells are implicated in biological processes. All transcriptome profiles are filtered for genes which display an FPKM value of at least 1.0 in both replicates of at least one biological condition. Differentially expressed genes are defined as those with an FDR of ≤0.05 and an absolute log2 fold change value of ≥0.6. **(A,B)** Percentage **(A)** and absolute count **(B)** of genes displaying differential expression between pairs of neural differentiation stages, divided into protein coding and lncRNA. Percentage values are calculated using the size of the transcript category as the denominator. **(C)** Dot plot of GO terms enriched in differentially expressed gene sets. Top 10 sets are selected in order of Benjamini-Hochberg adjusted *p*-values in each condition pair. Size of the dots indicate the number of differentially expressed genes associated with the GO term. **(D)** Scaled heatmap of the expression values of transcripts showing stage-specific expression according to the ROKU tissue specificity index. **(E)** Dot plot of GO terms enriched in stage specific gene sets. Top 10 sets are selected in order of Benjamini-Hochberg adjusted *p*-values in each condition pair. Size of the dots indicate the number of differentially expressed genes associated with the GO term.

Afterwards, we used differential expression analysis to understand which genes in the transcriptome showed significant changes between stages of differentiation. Our findings revealed that the iPSC samples show the highest amount of differentially expressed genes (DEG), coding and non-coding, both in percentage of gene category (Figure 3A) and number of genes (Figure 3B), and also throughout the differentiation late stages having a lower amount of DEGs between each other. Between stages, the highest percentage of DEGs is found in the iPSC/induction contrast, and the lowest percentage is in the differentiation/maturation contrast, indicating that the majority of the cell fate determination and differentiation happens in the induction and progenitor expansion stages. A full list of the DEGs in each contrast is available in Supplementary File S4.

We also examined the DEGs in each contrast to identify which biological processes they are most strongly associated with. Using GO enrichment, we identified the top 10 biological processes for each contrast (Figure 3C). As expected, the most significant terms are found between iPSCs and the other stages, with key terms including those associated with stemness, development and differentiation. Contrasts between later stages show lower significance, as a result of the lower number of DEGs found between the stages. Synapse formation mediating cell-cell communication between neurons is a complex process that is regulated by wide variety of molecules and transmembrane proteins (Südhof, 2018). It is worth noting that the existence of GO terms regarding synapse assembly and axon development between iPSCs and other conditions indicates the proper induction of iPSCs toward neurons. Among the DEGs associated with these GO terms, the semaphorin genes (SEMAs), which constitute a large family of secreted ligands and transmembrane proteins (Kumanogoh and Kikutani, 2004), show changes in expression in induction and subsequent differentiation stages. For instance, SEMA3B, which plays a role in axonal guidance and positioning of the brain anterior commissure, SEMA6B, which acts as a receptor in post-crossing commissural axon guidance (Moreno-Flores et al., 2003; Julien et al., 2005), SEMA3A, which has a role in dendritogenesis (Molofsky et al., 2014), and SEMA5A, which has a bifunctional role in axon development (Kantor et al., 2004), are upregulated during differentiation. In contrast, SEMA4D (Yamaguchi et al., 2012), which acts as an inhibitor of neuronal differentiation by promoting apoptosis, is downregulated in late stages of differentiation. Bone morphogenetic proteins (BMPs) form a large family of molecules and belong to the transforming growth factor-β (TGF-β) superfamily, which have critical roles in embryogenesis, neural induction, specification, and nervous system development (Mehler et al., 1997; Bond et al., 2012; Hegarty et al., 2013). Our results are consistent with previous studies, showing BMP signaling pathway ligands and receptors are crucial for neurogenesis. However, high levels of BMP7 and BMP4 expression were detected in the early induction stage, and increased levels of expression for several members of the BMP signaling pathway, such as BMP4, BMP6, and BMP1, were also observed across the late stages of differentiation, especially in the maturation stages. In addition, our results show the existence of BMP signaling pathway related genes among the most significant GO terms, such as connective tissue development, axon development, epithelial tube morphogenesis, and transmembrane receptor protein serine/threonine kinase signaling pathway, highlighting the importance of this signaling pathway in neuronal development. In early development stages expression of BMP4 is inhibited with Noggin to allow the neural induction (Thomsen, 1997). Reverse correlation between Noggin and BMP4 expressions in our results suggests that the gradual expression of BMP4 might play a vital role in iPSC-derived neuron differentiation. Of note, crosstalk between BMP and other signaling pathways, such as Wnt, SHH, and MAPK signaling pathway, in conjunction with extracellular matrix organization may govern the determination of cell decision (Compagnucci et al., 2014). The full list of enriched GO terms for each contrast is available in Supplementary File S5.

### 2.4 Identification and Annotation of Stage Specific Protein Coding and lncRNA Genes

As a follow-up to the identification of DEGs between stages, we identified genes with stage specific upregulation or downregulation during the entire process. As the DEG identification method only uses pairwise contrasts, and driver factors of cell fate specification and differentiation are transiently expressed or repressed (Semrau et al., 2017), it is vital to determine such stage specific expression patterns to act as markers of individual differentiation stages. To do so, we used the ROKU algorithm, a tissue specificity index used to determine which genes show increased or decreased expression in a stage specific manner across multiple samples, as described in the Section 4. After identifying protein coding genes with stage specific expression, we observed that the genes were divided into four clusters, as shown on the heatmap in Figure 3D. Cluster 1 comprised the genes downregulated in iPSCs compared to cells undergoing induction and differentiation, Cluster 2 comprised genes upregulated in iPSCs, while Cluster 3 and 4 were genes upregulated in the induction and maturation stages, respectively. The full list of genes in each cluster are available in Supplementary File S6. Similarly to Figure 3C, we also identified the top ten enriched GO terms for each cluster, according to adjusted *p*-value to determine the biological processes active during each stage (Figure 3E). Cluster 2, due to its large size, had the highest number of genes in its enriched terms, with 199 genes out of 2,314 in the “ribonucleoprotein complex biogenesis” gene set. In comparison, Cluster 1 only had a maximum of 27 out of 403 annotated genes in its top ten terms, in the “autophagy” and “process utilizing autophagic mechanism” gene sets, while cluster 3 and 4 both had 13 out of 140 and 159 annotated genes, respectively, in their top enriched GO terms (cilium assembly for cluster 3, extracellular matrix organization for cluster 4). In addition, the size of the gene set of the individual GO terms also affect the adjusted *p*-values, therefore genes with lower counts could be found to have lower adjusted *p*-values for their enrichment (Supplementary File S7). Based on the ROKU analysis, we found three distinct clusters consisting of iPSCs (clusters 1 and 2), progenitors (cluster 3) and mature neurons (cluster 4). In addition, stage specific functional terms were detected across the differentiation stages. The expression profile of iPSCs (clusters 1 and 2) and subsequent differentiated cells were clearly distinguishable and clustered by overall stage specific expression. In this regard, iPSC samples exhibited an expression profile typical of pluripotent stem cells, with NANOG, SALL4, and LIN28A all being upregulated, compared to other stages. Cluster 3 comprises genes which show increased expression in the early stage of differentiation (induction). The ROKU analysis for these genes shows they are primarily involved in the cilium assembly and organization process. Cilium is a unique cytoskeletal structure on the surface of most cells. It participates in signal transduction (Haycraft et al., 2005; Rohatgi et al., 2007), and plays an essential role during the early polarization of the neuroepithelium (Higginbotham et al., 2013), the expansion of the progenitor pool, formation of neural stem cells during nervous system development (Chizhikov et al., 2007; Spassky et al., 2008). Regulatory Factor X (RFX) transcription factors have been known to participate in the control of ciliogenesis by regulating many genes that play fundamental roles in cilia assembly, organization, and function (Thomas et al., 2010). Among these factors, RFX3 is a critical transcription factor in ciliogenesis and early brain development, where it indirectly regulates GLI3 and FGF8 to distribute neurons guidepost to morphogenesis (Benadiba et al., 2012). Our analyses demonstrated that high expression of RFX3 and GLI3 in progenitor cells appears to be informative of molecular cues throughout iPSC-derived neuron generation. In addition, any deficiency in the genes associated with ciliary causes several syndromes in humans. Unraveling of the gene network pattern during early brain development will provide an insight into the identification of the causes of such brain defects. Cluster 4 is obviously distinct, and includes genes with low expression in earlier stages of differentiation which increase at the maturation stage. The high expressions of LXN, C4A, and GAS6 during the maturation stage are consistent with previous studies, which showed a gradual elevation of LXN gene over the course of development (Arimatsu, 1994; Arimatsu et al., 1999; Arimatsu et al., 2009). In addition, the GAS6 gene promotes the survival of neurons, and its expression starts in later embryonic stages, remaining elevated in adults (Prieto et al., 1999). The ROKU analysis for these genes revealed that iPSC-derived neuron differentiation has the potential to mimic the *in vivo* developmental process.

### 2.5 Co-Expression Network Analysis and Functional Annotation of Differentiation Stage Associated lncRNAs

**FIGURE 4:**
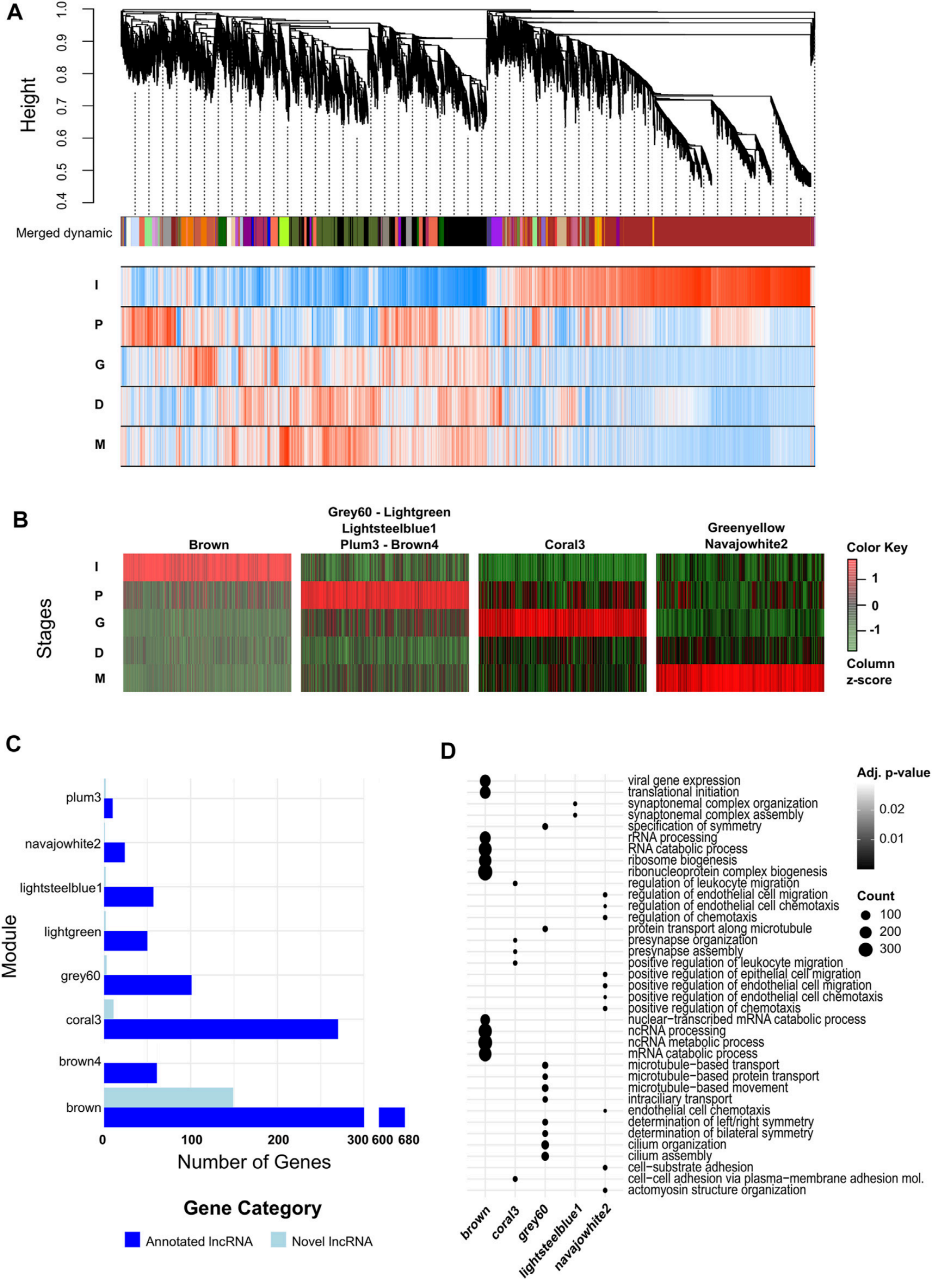
lncRNAs expressed during different stages of neural differentiation from iPSCs are associated with protein coding genes implicated in neural development biological processes. **(A)** WGCNA dendrogram and module affiliation graph of the transcriptome and association of gene expressions with biological conditions during neural differentiation. Each branch of the dendrogram represents a single gene expressed during neural differentiation. The colored bar under the dendrogram indicate the module the gene belongs in, with each color indicating a single module. The heatmap underneath the colored bar shows stage-module correlation levels, with red cells indicating positive correlation, blue cells indicating negative correlation, and darker colors indicating stronger correlation levels. **(B)** Scaled heatmaps of genes in modules showing strong association with single maturation stage (Pearson correlation coefficient ≥0.7). **(C)** Bar graphs indicating lncRNA module membership in clusters of interest categorized into annotated and novel lncRNAs. **(D)** Dot plot of GO terms enriched in gene clusters of interest. Up to 10 sets are selected in order of Benjamini-Hochberg adjusted *p*-values in each module. Size of the dots indicate the number of genes in the module associated with the GO term.

After the identification of both individual DEGs and stage specific protein coding genes, and the annotations of those sets, we further wanted to identify the genes working in tandem during each stage, in order to annotate the potential functions of novel or otherwise poorly annotated lncRNAs. Using WGCNA, we generated a dendrogram and gene/trait association heatmap to identify co-expressed gene modules (Figure 4A, Supplementary File S8). Once the modules were identified, we further used a module/trait association matrix, using differentiation stages as traits, to identify which modules were expressed with strong correlation with individual stages. We observed nine modules strongly correlated with a stage (*r* > 0.7 and *p*-value < 0.05). Five of the modules (lightgreen, brown4, grey60, lightsteelblue1, plum3) were associated with the induction stage (P). Two modules (greenyellow, navajowhite2) were associated with the maturation stage (M), while the iPSC (I) and progenitor (G) stages both had a single module associated with them (brown and coral3, respectively). No modules were strongly correlated with the differentiation stage (D) (Supplementary Figure S5). We also plotted the expressions of the genes found in these modules across the maturation process (Figure 4B). The genes in each module showed a significant increase in expression in the stage the module is associated with, indicating an accurate module—stage correlation analysis. We then observed the lncRNA membership of each of the seven modules, divided into annotated and novel lncRNAs, to identify how likely each module is to predict the behavior of lncRNAs (Figure 4C). Modules brown and coral3 had a high number of lncRNAs compared to the remaining modules, as well as a higher number of novel lncRNAs in particular. In particular, the module brown4 had no novel lncRNAs. We futher performed GO enrichment analysis on the modules to identify the main biological processes the modules are involved in (Figure 4D, Supplementary File S9). Five of the modules were enriched for at least one biological process. The brown module, associated with the iPSC stage, was enriched for terms associated with noncoding RNA regulation, ribosome formation, and gene transcription and translation. The modules grey60 and lightsteelblue1, associated with induction (P), were enriched for terms associated with synapse formation and cytoskeletal regulation, and the module coral3, associated with progenitor cells (G), was enriched for terms involved in cell-to-cell adhesion and presynapse assembly. Finally, the module navajowhite2, associated with the maturation stage (M), was enriched for terms associated with cellular migration and chemotaxis.

Following the analysis of modules, we performed hub gene identification in our modules. Hub genes in networks have high connectivity to the other genes in the network, and are likely to be critical actors in the activity of those networks (van Dam et al., 2018). As a result, identifying any lncRNAs as hub genes during the iPSC-derived neuron differentiation process would allow us to narrow down our list of targets for future research. Out of 16,699 genes found in the identified modules, 5,163 were considered to be hub genes (kME > 0.90, Supplementary File S10). 89 of the hub genes were novel lncRNAs, and 707 were annotated lncRNAs. Furthermore, 55 of the novel lncRNA hub genes, and 257 of the annotated lncRNA hub genes were found in modules showing strong correlation with individual maturation stages.

Recent studies have reported that lncRNAs are involved in the regulation of cellular processes in mammalian development and disease. Nevertheless, many lncRNAs have unknown biological functions. Our WGCNA and GO analyses predict possible roles for lncRNAs in a wide range of biological processes, such as ncRNA processing, establishment of protein localization to organelles, sensory perception of bitter taste, cilium assembly, microtubule-based movement, neural tube development, and axonogenesis. Additionally, Our findings show that the AC006062.1 and AC025280.3 lncRNAs were upregulated in the differentiation and maturation stages. The high expression of these lncRNAs was accompanied with the upregulation of coding genes such as C4A and C4B in the maturation stage, both of which are hub genes (kME > 0.94) in a module associated with maturation. Furthermore, the alteration of LINC00261, C4A, and C4B following valproic acid treatment in motor neurons (Yoshida et al., 2015), as well as the clustering of AC025280.2 with neuron-related genes (Tenjin et al., 2020), suggest the possible role of these genes in neuron development. Additionally, while the expression and function of GAS6-DT in neurons and the brain remain unclear, there is evidence that GAS6-DT is involved in the upregulation of GAS6 gene expression in melanomas (Wen et al., 2019). GAS6 is also a protein-coding hub gene associated with maturation (kME > 0.90), with roles in the central nervous system (CNS) (Goudarzi et al., 2016), and highlighting a similar crosstalk between GAS6 and GAS6-DT can influence neuron development and maturation in the context of iPSC-derived neuron generation. Our results also revealed the upregulation of MIRLET7BHG (kME > 0.92) in the maturation stage of differentiation. This data is in agreement with previous reports that demonstrated the expression of MIRLET7BHG in various tissues, including the brain (Sauvageau et al., 2013). In addition, LINC00842 and LINC00857 are lncRNAs with unknown function in the brain, but LINC00842 downregulation was detected in the lung adenocarcinoma sample, compared to healthy tissue (Ding et al., 2018). In addition, LINC00857 is one of the lncRNAs dysregulated in lung cancer (Wang et al., 2016; Wang et al., 2020), and it regulates biological processes such as tumor growth, proliferation, motility, and the invasion capacity of lung cancer, in addition to acting as an oncogene in liver (Xia et al., 2018), bladder (Dudek et al., 2018), gastric (Pang et al., 2018), and esophageal cancer models (Su et al., 2019). In our analyses, the upregulation of LINC00842 and LINC00857 throughout differentiation suggests that LINC00857 might have roles in the biological response of cells during the maturation of neuron cells. In addition to the novel lncRNA candidates in our results, we found upregulation of expression of lncRNAs known to be active during neurogenesis identified as hub genes in our study, such as MALAT1 (kME > 0.96) (Bernard et al., 2010; Lipovich et al., 2012) and TUNA (kME > 0.90) (Lin et al., 2014). Thus, these results might be used to understand the cell compositions and differentiation stages of iPSC-derived neuronal cultures and discovery of novel markers throughout brain development.

## 3 Conclusion

Understanding the transcriptome is a critical step in study of the differentiation process in multicellular organisms, as changes in the transcriptome are what allows the large variety of cells required for the formation of a multicellular organism to arise from undifferentiated cells with a shared genome. While the protein coding transcripts play an important role in cell differentiation and fate determination, our understanding of the non-coding transcriptome and its role in these processes is as of yet incomplete. As a large percentage of the genomes of higher order eukaryotes is made up of non-coding genes with functional roles in chromatin organization and the regulation of gene expression, an in-depth analysis of the full transcriptome, as opposed to the coding transcriptome, is crucial in studying processes such as the formation of neurons from undifferentiated stem cells. Such in-depth analysis of the transcriptome has been made available in the last two decades by advent of high-throughput sequencing technologies, such as RNA-seq, as well as the computational tools used in processing the sequencing data.

Here, we presented a detailed analysis of the transcriptome during multiple stages of iPSC-derived neuronal differentiation. We included comparisons and contrasts between the stages, and identify biological processes enriched during specific stages. We provided an overview of the most significant terms and co-regulated gene modules, as well as a comparison of our findings to previously established literature on cell differentiation and proliferation. In order to provide a valuable resource for future research on neural development and neuron differentiation, we further included in-depth lists of differentially expressed or stage-specific genes, and co-expressed gene modules, as well as enriched GO terms in each of these categories. Crucially, we have also provided detailed data regarding the expressions of lncRNAs during iPSC-derived neuronal differentiation, and potential differentiation-affiliated biological processes they are implicated in. The comprehensive map of the coding and non-coding transcriptome during neuronal differentiation is of great importance to future research in both developmental biology and neuroscience.

## 4 Materials and Methods

### 4.1 iPSC Expansion

Two healthy human iPSC lines from two independent donors, represented as iPSC line WT1 (home-made) and WT2 (Cat No. #ASE-9202, Applied StemCell Inc.) were cultured and maintained as previously described (Akbari et al., 2019b). iPSCs expanded on hESC-qualified Matrigel matrix basement membrane (cat no: #354277, Corning) with mTeSR1 medium (cat no: #SC-05850, Stem Cell Technologies). Cells were passaged once a week with a 1:6 ratio, and culture medium was changed every other day following sub-culturing.

### 4.2 Neural Induction and Expansion of Neural Progenitor Cells

We used STEMdiff^™^ Neural System to generate iPSC-derived neuron cells. Production procedure comprise mainly the induction/generation, expansion, differentiation and maturation steps. In all steps of differentiation, cells were cultured in a monolayer culture system, and we did not isolate or enrich cells according to their surface markers while sub-culturing the cells. iPSCs were harvested from the mTeSR1 culture, and plated on matrigel coated plates at 200,000 cells/cm^2^ in neural induction medium (NIM) supplemented with SMADi (cat no: # 08581, Stem Cell Technologies) and 10 µM Y-27632 (cat no: # 72302, Stem Cell Technologies) for 9 days. Afterwards, the generated NPCs in NIM were sub-cultured for two additional passages before starting differentiation as recommended in the manufacturer's protocol. To this end, the NPCs were detached with Accutase (cat no: # 07922, Stem CellTechnologies), seeded at 1.25 × 10^5^ cells/cm^2^ on Matrigel coated plates, and expanded in neural progenitor medium (NPM) (cat no: # 05833, Stem Cell Technologies) for the next 20 days. The samples were collected on day 0 (pluripotent), days 9–12 (induced progenitor), and days 25–28 (expanded progenitor).

### 4.3 Neural Differentiation and Maturation

To generate mature neuron cells, two more passages were performed during days 25–28 (first day of differentiation) and days 32–35 (first day of maturation), respectively. Neural differentiation medium (NDM) (cat no: # 08500, Stem Cell Technologies) was used to generate neuronal precursors from iPSC-derived NPCs. On days 25–28, NPCs were placed at a density of 4 × 10^4^ cells/cm^2^ on matrigel coated plates. After overnight incubation at 37°C and 5% CO_2_ in the incubator, the culture medium was fully refreshed with NDM, and the process continued for 7 days. On days 32–35, generated neural precursor cells were passaged and plated at a density of 4 × 10^4^ cells/cm^2^ on matrigel coated cell culture plates. The culture medium was switched to neural maturation medium (NMM) (cat no: # 08510, Stem Cell Technologies) on the following day, and the cells were incubated at 37°C and 5% CO_2_ in the incubator for at least 1 week. The samples were collected on days 32–35 (differentiation) and days 45–47 (maturation).

### 4.4 Immunofluorescence Staining

Characterization of the generated cells during differentiation stages was performed using immunofluorescence staining, as previously described (Akbari et al., 2019b; Karagonlar et al., 2020). Briefly, the cells were fixed in %4 paraformaldehyde (PFA; cat no: # 158127, Merck) for 20 min at room temperature, washed three times with 1× PBS, then permeabilized using 0.5% TritonX (cat no: #28313, Thermo Fisher Scientific). After 2 hours, blocking staining was carried out using the following primary antibodies: OCT3/4 (cat no: # 75463S, Cell signaling), PAX6 (cat no: # 60433S, Cell signaling), NESTIN (cat no: # 33475S, Cell signaling), GFAP (cat no: # 12389T, Cell signaling) and β-TUBIII (cat no: # 4466S, Cell signaling). Slides were visualized using a confocal microscope (cat no: # LSM880, Zeiss).

### 4.5 RNA Extraction and Sequencing

Total RNA was isolated using the Nucleospin RNA II kit (Macherey-Nagel, Düren, Germany) according to the manufacturer’s instructions. The RNA concentration was measured *via* NanoDrop (Thermo Fisher Scientific), and the quality was assessed using Agilent Bioanalyzer. RNA sequencing was performed at EMBL GeneCore. Briefly, the samples were prepared using NEBNext^®^ rRNA Depletion Kit (Human/Mouse/Rat) and the NEBNext^®^ Ultra^™^ II Directional RNA Library Prep Kit for Illumina^®^ to generate strand-specific RNA libraries. We started with 250 ng of total RNA as input, adaptor dilution was 1:5, and we used nine cycles for the PCR enrichment of adaptor ligated DNA. Then 5-plex pools were prepared equimolarly and sequenced in a NextSeq 500 system with 40 pair-end read model. The samples were sequenced to an average depth of 100 million reads per sample.

### 4.6 Gene Expression Measurement

Paired-end RNA-seq reads were aligned against the GENCODE GRCh38 human genome assembly (Release 34, obtained from https://www.gencodegenes.org/) using HISAT2 (version 2.1.0) (Kim et al., 2015) with the parameters “-p 36 --dta -× -1 -2 -S”. The resulting SAM alignment files were converted to BAM binary files and sorted and indexed using SAMtools utilities (version 1.9) (Li et al., 10002009). The alignment files were used to calculate the total expression levels of gene transcripts (including all known isoforms) using the featureCounts function of the R package Rsubread (version 2.4.0) (Liao et al., 2019) with the following parameters: “files = {infile.bam}, annot.ext = "{infile.gtf}", isGTFAnnotationFile = T, GTF.featureType = “exon”, GTF.attrType = “gene_id”, useMetaFeatures = T, countMultiMappingReads = T, isPairedEnd = T, nthreads = numParallelJobs.” Identified transcripts were annotated using the GENCODE GRCh38 human transcriptome annotations (Release 34). Following expression quantification, we removed transcripts that did not have an expression of ≥1 FPKM (fragments per kilobase of transcript per million reads) in both replicates of at least one biological condition to improve detection sensitivity of differentially expressed genes.

### 4.7 Identification of Novel lncRNA Candidates

A number of filters were applied to transcripts that were not annotated by our reference transcriptome assembly in order to identify high confidence novel lncRNA candidates. Transcripts coded “u,” “x,” “o,” and “i” by StringTie were selected as the initial candidate pool, which signify transcripts aligned to intergenic regions, to the antisense strand of known genes, to the sense strand of known genes with partial exonic overlap, and to the intronic regions of known genes, respectively. We further selected only transcripts longer than 200 nucleotides for the subsequent analyses. The remaining transcripts were analyzed to identify those with high coding potential and an ORF coding for longer than 100 aminoacids with TransDecoder (https://github.com/TransDecoder/TransDecoder) the “TransDecoder.LongOrfs -t” command, which were removed from the analysis. The remaining transcripts were aligned against the SwissProt manually curated protein sequence database (version 2017_08) to identify any protein domain homology, as is found in pseudogenes, using a local installment of blastx, with an E-value cutoff of 0.01 (blastx -evalue 0.01). The remaining transcripts were aligned against the Rfam database of RNA families (version v12.1) (Nawrocki et al., 2015) with the Infernal cmscan program, with an E-value cutoff of 0.01 (−E 0.01), in order to identify any housekeeping RNAs. Finally, transcripts with a human-specific coding probability of 0.8 or higher, as identified by CPAT (version 1.2.4) (Wang et al., 2013) were considered to be protein coding and removed from the list of novel lncRNA candidates (Hudson et al., 2019).

Independent iPSC-derived neuronal differentiation datasets were used to inspect the expressions of identified lncRNAs. A total of 52 raw RNA-sequencing libraries were downloaded from the Sequence Read Archive (SRA) database (Leinonen et al., 2011), pertaining to previous studies of iPSC-derived neurons (SRA Accession #s: SRP238174, SRP266877). The libraries were downloaded in FASTQ format using the fastq-dump utility of the SRA Toolkit (v.2.9.0), with the following parameters: “*--gzip--skip-technical--readids--dumpbase--clip--split-3.”* The libraries were then processed and quantified using the same methods as described above. Genes were considered consistently expressed in a dataset if it had an expression of ≥1 FPKM of more than 50% of the samples in at least one biological condition in the dataset.

### 4.8 Differential Expression Analysis

The differential expression statuses of protein-coding and lncRNA transcripts were analyzed using the R package edgeR (version 3.32.0) (Robinson et al., 2010). All condition pairs were examined to identify transcripts that are upregulated in one condition compared to the second, for a total of 10 combinations. Count data of the transcripts were normalized using the trimmed mean of M-values method (TMM), which were then fitted to a generalized linear model (GLM). Afterwards, the estimateDisp and glmFit functions were used to calculate the contrast statistics for the condition pairs. Genes were considered differentially expressed between two conditions if they had an adjusted *p*-value (FDR) of 0.05 or lower, and an absolute log2 (fold change) value of 0.6 or higher.

### 4.9 Identification of Stage Specific Expression Patterns

To identify which protein coding genes had stage-specific expression patterns, whether upregulation or downregulation, and which stages of the differentiation process they were specific to, we used the ROKU function of the R package TCC (version 1.24.0) (Sun et al., 2013). ROKU is an algorithm that analyzes the expression levels of a gene across multiple samples, whether a time-course series or discrete biological conditions, and identifies whether any of the values are outliers (Kadota et al., 2006). It then marks them with a 1 if it is upregulated in a sample compared to the other samples, and −1 if it is downregulated. Samples with nonspecific expression patterns are marked 0. Genes with consistently low expression (<1 FPKM in at least one replicate of all stages) were also marked 0 to avoid noise.

### 4.10 Weighted Gene Co-Expression Network (WGCNA) Analysis

To examine the potential coregulatory relationships between genes expressed during the differentiation stages, we used the R package WGCNA (version 1.69) (Langfelder and Horvath, 2008) to perform weighted gene coexpression network analysis using the FPKM expression values of transcripts and created a coexpression network. We set a soft thresholding power of 18 for the correlation network formed prior to the coexpression analysis, as recommended by WGCNA for our experimental design. The Dynamic Tree Cut algorithm of WGCNA was then applied to a hierarchical clustering of the genes using the average linkage method to identify clusters of co-expressed genes, or gene modules. A minimum module size of 30 genes was set to avoid excessive noise in module determination. The correlation of each module eigengene with the differentiation stage is calculated and significant modules associated with the stages were determined (*r* > 0.7 and *p*-value < 0.05). The hub genes in each module were calculated using intramodular connectivity scores (kME). Genes with a kME of 0.90 or higher were considered to be hub genes.

### 4.11 Statistical Analysis and Graphical Representation

We used the R statistical computation environment (version 3.6.0) for all analysis and visualization purposes. Functions of the stats package or the base R installation were used for most statistical analysis, either directly or by other packages, including hclust for Euclidean hierarchical clustering of genes and samples, prcomp for identification of principal components of the expression matrix, and cor.test for correlation calculation. clusterProfiler (version 3.18.0) (Yu et al., 2012) was used to identify and visualize the enrichment of GO terms in sets of genes of interest. Pheatmap (https://cran.r-project.org/web/packages/pheatmap/index.html) (version 1.0.12) was used with row-wise scaling of data to visualize the Z-score values of genes during differentiation. Native WGCNA functions were used to visualize the coexpression network dendrogram, module colors, and relationships of such with specific biological conditions. ggplot2 (https://cran.r-project.org/web/packages/ggplot2/index.html) (version 3.3.2) was used for all other visualization.

## Data Availability

The datasets presented in this study can be found in online repositories. The RNA-seq datasets are deposited into the Sequence Read Archive (SRA), with accession number SRP304457.

## Supplementary Material

Supplementary Figure S1 Bar plot displaying the percentages of read counts of expressed transcripts in stages of iPSC-derived neuronal differentiation. lncRNAs include annotated and novel intergenic lncRNAs, sense overlapping lncRNAs, intronic RNAs, and antisense RNAs. Other RNAs include any transcript that is not protein coding or in the lncRNA category, including miRNAs, piRNAs, siRNAs, snRNAs, and snoRNAs.

Supplementary Figure S2 Violin plot displaying the distributions of log2- transformed FPKM expression values of protein coding and lncRNA genes during iPSC-derived neuronal differentiation. Horizontal lines within the violin shapes indicate the median log2-transformed FPKM value.

Supplementary Figure S3 Scaled heatmap of the log2(FPKM + 1) expression values of novel lncRNAs identified in iPSC-derived neuronal differentiation process consistently expressed in the samples from the SRP238174 dataset (>1 FPKM in at least 50% of one timepoint). Purple cells indicate increased expression compared to the mean, while green cells indicate decreased expression. Dataset originally described by Burke et al. (2020).

Supplementary Figure S4 Scaled heatmap of the log2(FPKM + 1) expression values of novel lncRNAs identified in iPSC-derived neuronal differentiation process consistently expressed in the samples from the SRP266877 dataset (>1 FPKM in at least 50% of one timepoint). Purple cells indicate increased expression compared to the mean, while green cells indicate decreased expression. Dataset originally described by Solomon et al. (2021).

Supplementary Figure S5 Module – trait relationship heatmap of coexpressed gene modules and iPSC-derived neuronal differentiation stages. Top number in each cell indicates the Pearson correlation between the module expression and the differentiation stage. Bottom number (in parentheses) indicates the *p*-value of the correlation. Red cells indicate a positive correlation, while green cells indicate a negative correlation.

Supplementary File S1 GTF file of transcripts identified as high-confidence lncRNAs.

Supplementary File S2 Expression matrix of novel lncRNA candidates in SRP238174.

Supplementary File S3 Expression matrix of novel lncRNA candidates in SRP266877.

Supplementary File S4 List of differentially expressed genes in each contrast between conditions.

Supplementary File S5 List of biological process GO terms enriched in the DEGs of each contrast.

Supplementary File S6 List of genes that are stage-specifically expressed during differentiation.

Supplementary File S7 List of biological process GO terms enriched in stage-specifically expressed gene clusters.

Supplementary File S8 List of gene memberships of co-expressed gene modules.

Supplementary File S9 List of biological process GO terms enriched in co-expressed gene modules.

Supplementary File S10 List of identified hub genes and their module memberships.
