# Supplementary material for: Transcriptome Dynamics of Human Neuronal Differentiation From iPSC
Source: Front Cell Dev Biol. 2021 Dec 14;9:727747. doi: 10.3389/fcell.2021.727747 (PMC8712770; doi:10.3389/fcell.2021.727747)
Supplement: Supplementary file 3 [file Image5.pdf]

**S5**

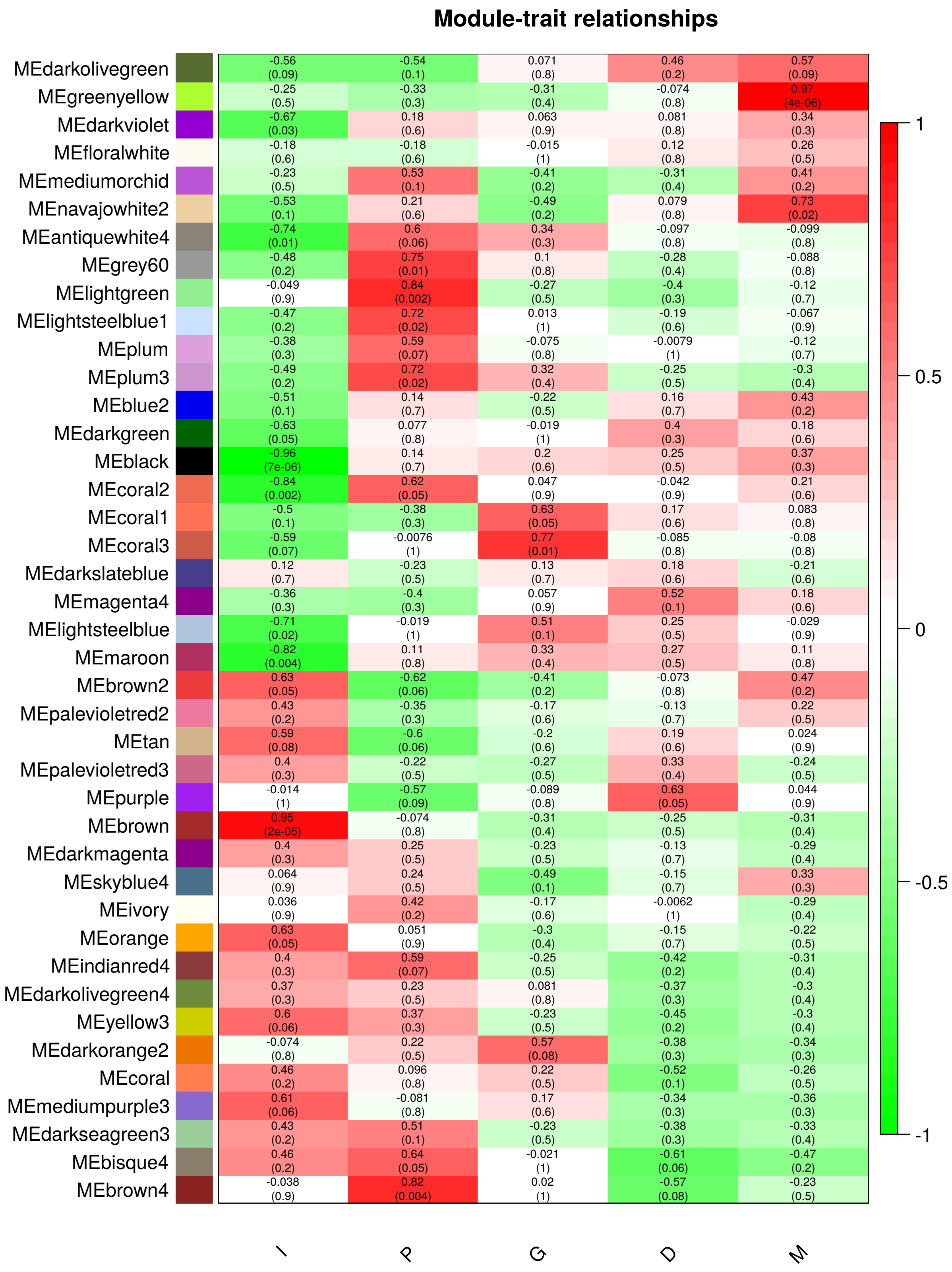


**Supplementary Figure 5 –** Module – trait relationship heatmap of coexpressed gene modules and iPSC-derived neuronal differentiation stages. Top number in each cell

indicates the Pearson correlation between the module expression and the differentiation stage. Bottom number (in parentheses) indicates the p-value of the correlation. Red cells indicate a positive correlation, while green cells indicate a negative correlation.
