# Supplementary material for: Transcriptome Dynamics of Human Neuronal Differentiation From iPSC
Source: Front Cell Dev Biol. 2021 Dec 14;9:727747. doi: 10.3389/fcell.2021.727747 (PMC8712770; doi:10.3389/fcell.2021.727747)
Supplement: Supplementary file 6 [file Image2.pdf]

S2

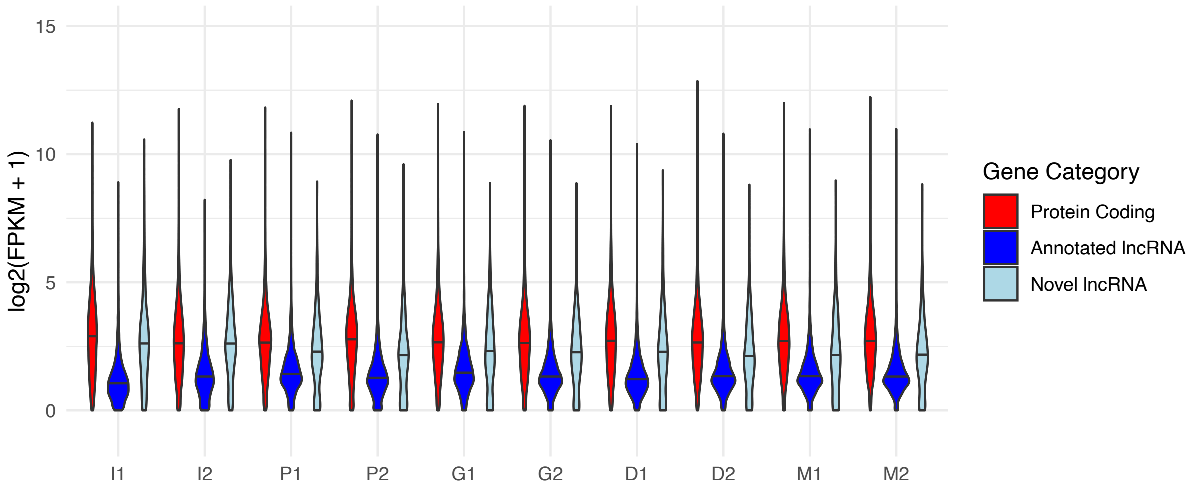

Supplementary Figure 2 – Violin plot displaying the distributions of log2-transformed FPKM expression values of protein coding and lncRNA genes during iPSC-derived neuronal differentiation. Horizontal lines within the violin shapes indicate the median log2-transformed FPKM value.
