# Supplementary material for: Transcriptome Dynamics of Human Neuronal Differentiation From iPSC
Source: Front Cell Dev Biol. 2021 Dec 14;9:727747. doi: 10.3389/fcell.2021.727747 (PMC8712770; doi:10.3389/fcell.2021.727747)
Supplement: Supplementary file 8 [file Image3.pdf]

S3

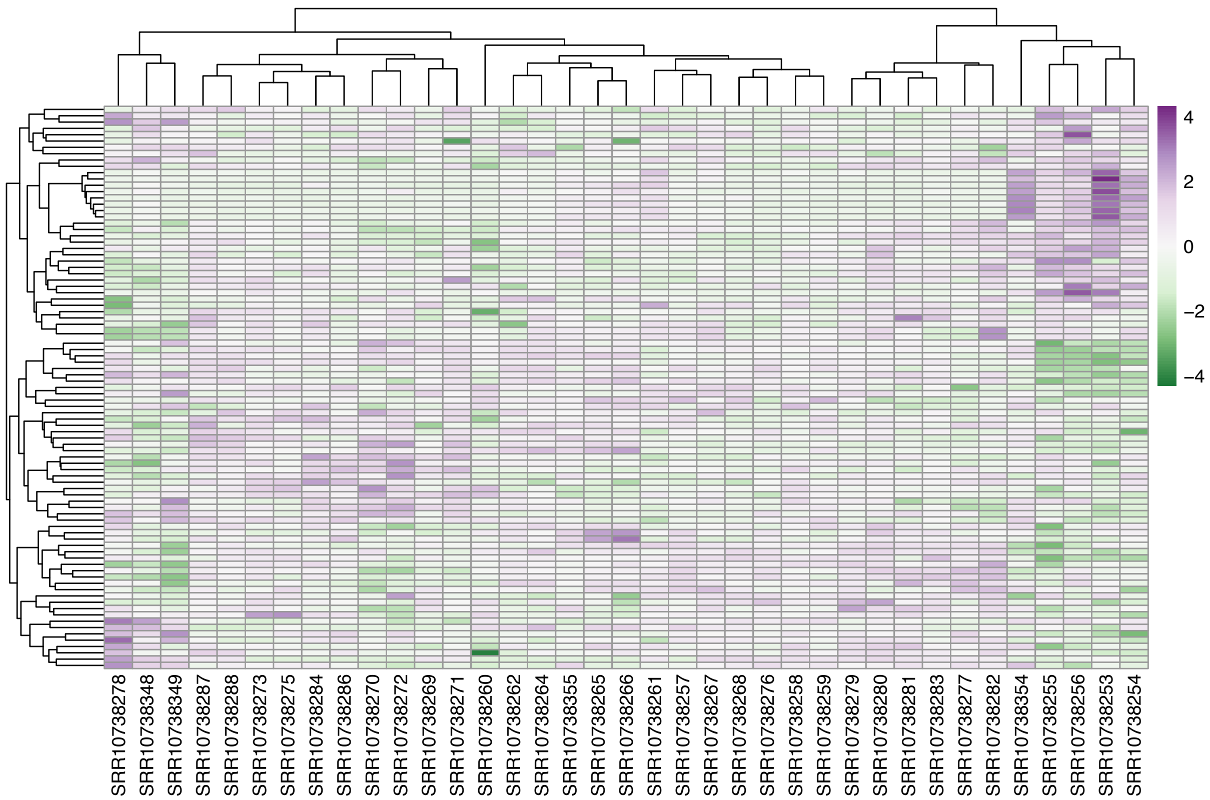

Supplementary Figure 3 – Scaled heatmap of the log2(FPKM + 1) expression values of novel lncRNAs identified in iPSC-derived neuronal differentiation process consistently expressed in the samples from the SRP238174 dataset (> 1 FPKM in at least 50% of one timepoint). Purple cells indicate increased expression compared to the mean, while green cells indicate decreased expression. Dataset originally described by Burke, et al. (Nature Communications, 2020).
