# Supplementary material for: Transcriptome Dynamics of Human Neuronal Differentiation From iPSC
Source: Front Cell Dev Biol. 2021 Dec 14;9:727747. doi: 10.3389/fcell.2021.727747 (PMC8712770; doi:10.3389/fcell.2021.727747)
Supplement: Supplementary file 15 [file Image1.pdf]

S1

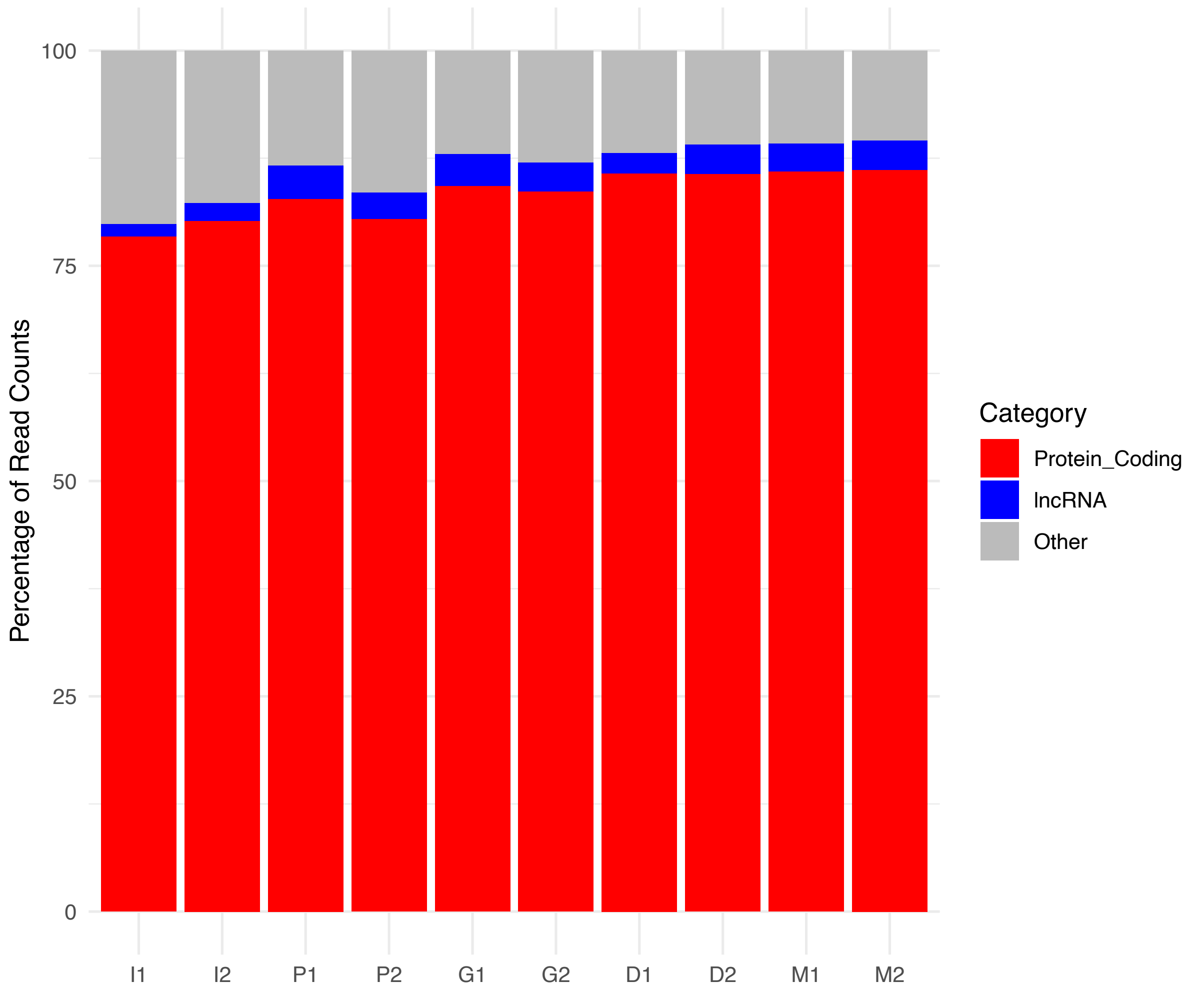

Supplementary Figure 1 – Bar plot displaying the percentages of read counts of expressed transcripts in stages of iPSC-derived neuronal differentiation. lncRNAs include annotated and novel intergenic lncRNAs, sense overlapping lncRNAs, intronic RNAs, and antisense RNAs. Other RNAs include any transcript that is not protein coding or in the lncRNA category, including miRNAs, piRNAs, siRNAs, snRNAs, and snoRNAs.
